# Development of Bioceramic Bone-Inspired Scaffolds Through Single-Step Melt-Extrusion 3D Printing for Segmental Defect Treatment

**DOI:** 10.3390/jfb16100358

**Published:** 2025-09-23

**Authors:** Aikaterini Dedeloudi, Pietro Maria Bertelli, Laura Martinez-Marcos, Thomas Quinten, Imre Lengyel, Sune K. Andersen, Dimitrios A. Lamprou

**Affiliations:** 1 School of Pharmacy, Queen’s University Belfast, 97 Lisburn Road, Belfast BT9 7BL, UK; adedeloudi01@qub.ac.uk; 2 Wellcome-Wolfson Institute for Experimental Medicine, School of Medicine, Dentistry and Biomedical Sciences, Queen’s University Belfast, 97 Lisburn Road, Belfast BT9 7BL, UK; 3 Johnson & Johnson Innovative Medicine, Oral Solids Development, Research & Development, Turnhoutseweg 30, 2340 Beerse, Belgium

**Keywords:** 3D printing, polycaprolactone, bioceramics, biocompatibility, implantable scaffolds

## Abstract

The increasing demand for novel tissue engineering (TE) applications in bone tissue regeneration underscores the importance of exploring advanced manufacturing techniques and biomaterials for personalised treatment approaches. Three-dimensional printing (3DP) technology facilitates the development of implantable devices with intricate geometries, enabling patient-specific therapeutic solutions. Although Fused Filament Fabrication (FFF) and Direct Ink Writing (DIW) are widely utilised for fabricating bone-like implants, the need for multiple processing steps often prolongs the overall production time. In this study, a single-step melt-extrusion 3DP technique was performed to develop multi-material scaffolds including bioceramics, hydroxyapatite (HA), and *β*-tricalcium phosphate (TCP) in both their bioactive and calcined forms at 10% and 20% *w*/*w*, within polycaprolactone (PCL) matrices. Printing parameters were optimised, and physicochemical properties of all biomaterials and final forms were evaluated. Thermal degradation and surface morphology analyses assessed the consistency and distribution of the ceramics across the different formulations. The tensile testing of the scaffolds defined the impact of each ceramic type and wt% on scaffold flexibility performance, while in vitro cell studies determined the cytocompatibility efficiency. Hence, all 3D-printed PCL–ceramic composite scaffolds achieved structural integrity and physicochemical and thermal stability. The mechanical profile of extruded samples was relevant to the ceramic consistency, providing valuable insights for further mechanotransduction investigations. Notably, all materials showed high cell viability and proliferation, indicating strong biocompatibility. Therefore, this additive manufacturing (AM) process is a precise and fast approach for developing biomaterial-based scaffolds, with potential applications in surgical restoration and support of segmental bone defects.

## 1. Introduction

Numerous strategies in orthopaedic surgery have been applied to effectively address segmental bone defects, such as minimally invasive surgery, arthroscopy, and medical devices. The complex and dense nature of bone tissue, as well as its deep anatomical site, pose significant challenges that hinder the feasibility, efficiency, and duration of new bone formation. Conventional treatments, such as the use of allografts and autografts, remain widely utilised. The former carries the risk of immune rejection, while the latter demands a second surgical site and extended recovery time [1]. On the other hand, tissue engineering (TE) presents a promising technique for musculoskeletal tissue restoration, leveraging the unique combination of biomaterials, cells, and growth factors [2,3]. This approach involves the incorporation of additive manufacturing (AM) techniques, such as 3D printing (3DP) and bioprinting, to produce anatomically precise, functional bone-resembling structures [4,5,6]. AM facilitates the use of versatile materials (e.g., polymers, cells [7] and biologically active molecules [8]), hence, the creation of biofunctional scaffolds, capable of integrating with the host tissue. Therefore, TE focuses on the development of biocompatible scaffolds that minimise the risk of immune rejection and eliminate immunosuppressive medications, enhance bioactivity to regulate bone repairment, and facilitate their biodegradability, avoiding the necessity for reoperation [9,10].

Material-extrusion 3DP techniques such as Fused Filament Deposition (FFF) and Direct Ink Writing (DIW) are extensively employed in biomedical applications for bone tissue regeneration. These additive manufacturing (AM) methods have significantly contributed to the fabrication of scaffolds that closely mimic the structural and porosity characteristics of the bone, owing to their feasibility in utilising ceramic-based materials (e.g., bioactive or bioinert ceramics, ceramic composites), their simplicity and cost-effectiveness [11]. However, limitations such as the multi-step process, regarding pre-formulation (e.g., solvent mixing, use of polymeric binders/gelling agents, etc.) and post-processing steps (e.g., sintering, solvent debinding, polymeric coating) [12,13,14], impede the incorporation of biologically and thermally sensitive components (e.g., proteins, drugs) during the printing process. Furthermore, the sintering process can compromise the structural integrity of the scaffolds and adversely affect the porosity precision and microporous architecture, thereby limiting their application for complex biological environments [11]. Thus, the involvement of numerous variables in a multi-step manufacturing process not only may complicate final product quality characterisation but also may constrain direct clinical translation and prolong the overall treatment duration.

Contrarily, direct melt-extrusion printing enables the fabrication of multi-material 3D structures within a single-step manufacturing process, ensuring both material uniformity and shape fidelity (SF) [15]. This single-step 3DP approach represents an emerging manufacturing (EM) technique that involves straightforward stages: (a) computer-aided design (CAD) modelling, (b) filament extrusion, and (c) layer-by-layer deposition within a consolidated in-house system [16]. This enables the precise fabrication of structures with customised dimensions, internal patterns, and material composition for patient-specific tissue-like architectures. Specifically, in musculoskeletal scaffold fabrication, the meticulous material selection in combination with the precise design of structure may enhance their mechanotransduction [17,18,19], influence vascularisation and cellular activity, ultimately supporting functional tissue integration and bone repair [20]. The simplicity of this process is also appealing for healthcare facilities, enabling the creation of patient-centred treatments. Lastly, as this technology employs a single melting step for producing micro-scale fibres, it subjects materials to only one thermal cycle, thereby enhancing the feasibility of material reuse characterising the process as a sustainable manufacturing technique [21].

The selection of biomaterials for fabricating bone substitute scaffolds is primarily based on their osteoconductive and osteoinductive properties and their ability to support the tissue sufficiently. A diversity of materials and their composites have been examined for bone TE applications, including natural (e.g., collagen [22], chitosan [23], GelMA [24]) or synthetic polymers (e.g., poly(*ε*-caprolactone) (PCL) [25], poly(ethylene glycol) (PEG), poly(lactic-co-glycolic acid) (PLGA)), inorganic materials (e.g., hydroxyapatite (HA [26]), *β*-tricalcium phosphate (TCP)) and biological matrices such as extracellular matrix (ECM) derivatives or cells sheets (e.g., mesenchymal stem cells—MSCs, human umbilical vein endothelial cells—HUVEC, osteoblasts) [1]. Among these, PCL, a hydrophobic, semi-crystalline, thermoplastic synthetic polymer, exhibits a slow biodegradability rate and notable biocompatibility. It is extensively employed in bone TE owing to its exceptional ductility, which facilitates tissue flexibility and increases permeability. This, consequently, enhances vascularisation and modulates cellular processes such as proliferation and differentiation [27]. Moreover, calcium phosphates, such as HA and TCP enhance the surface roughness of scaffolds, contributing to cell attachment, proliferation, and osteogenic differentiation [28]. Their intrinsic bioactivity promotes the adhesion and maturation of bone-forming cells, while their mechanical reinforcement increases stiffness, abrasion resistance, and tear strength, reducing susceptibility to mobility-induced mechanical stress [29]. Consequently, the combination of biopolymers and bioceramics not only modulates the surface morphological properties but also improves the mechanical integrity of scaffolds, aiming towards higher tissue support while facilitating its reconstitution.

The aim of this study was to fabricate bio-inspired 3D-printed implants for potential application in bone tissue regeneration (BTR), specifically to enhance post-operative restoration. The selected material combinations of PCL, HA and TCP were designed to improve scaffold stiffness for structural support while retaining an elastic profile to facilitate surgical insertion. Moreover, a single-step 3DP process was developed, facilitating the efficient utilisation of reduced material volumes, thereby obviating post-processing scaffold treatment steps and enabling the fabrication of final products with tailored dimensions and infill patterns, allowing for examination and further modulation of the mechanical behaviour of the extruded composites. Surface morphology and elemental composition analyses were performed to assess the composition and homogeneity of the scaffolds. In vitro cell studies were implemented to examine the cytocompatibility and potential cytotoxic effects, ensuring the suitability of the materials for biomedical applications.

## 2. Materials and Methods

### 2.1. Materials

Polycaprolactone (PCL) (MW: 50 kDa, particle size < 600 μm) was obtained by Polysciences Inc.^©^ (Warrington, PA, USA), hydroxyapatite (HA) (nanoXIM·HAp202, MW: 1004.6, d_50_: 5.0 ± 1.0 μm), calcined hydroxyapatite (HAc) (nanoXIM·HAp602) (MW: 1004.6, d_50_: 5.0 ± 1.0 μm), *β*-tricalcium phosphate (TCP) (nanoXIM·TCP200, MW: 310.09, d_50_: 5.0 ± 2.0 μm) and calcined *β*-tricalcium phosphate (TCPc) (nanoXIM·TCP600) (MW: 310.09, d_50_ ≤ 15.0 μm), were donated by FLUIDINOVA, S.A. (Maia, Portugal) (Table S1). Primary Human Pulmonary Fibroblasts (HPF) were purchased by PromoCell^®^ GmbH (Heidelberg, Germany), Dulbecco’s Modified Eagle Medium (DMEM), Fetal Bovine Serum (FBS), Formaldehyde, 4% in PBS, and DAPI (4′,6-diamidino-2-phenylindole) were supplied by ThermoFisher Scientific Inc. (Horsham, UK). Phosphate-buffered saline (PBS) tablets were obtained from Sigma Aldrich^©^ (Steinheim, Germany).

### 2.2. Methods

#### 2.2.1. Design and Development of Polymeric-Ceramic Composite Scaffolds

In this study, 8 distinct polymeric-ceramic formulations were designed for the fabrication of 3D-printed implants (Table 1). PCL was selected as the primary matrix material due to its biodegradability, lipophilic nature, and elastic behaviour. Four different bioceramics were utilised—HA, HAc, *β*-TCP and TCPc—to enhance the stiffness of the scaffolds. These specific ceramics were selected based on their small median particle size (d_50_), which is suggested to facilitate efficient extrudability during the melt-extrusion process, and their Ca/P ratio, which closely resembles that of natural HA. HA and TCP were chosen due to their bioactive nature, while HAc and TCPc (calcined ceramics) were included to gain a better understanding of the ceramic’s influence on the final product’s mechanical properties. The optimal wt% ceramic concentrations were determined using a mixture design, beginning with an initial ratio of 95:5 (wt%), with incremental increases of 5 wt% in each subsequent step, establishing 20% *w*/*w* as the maximum amount that could be efficiently incorporated into the polymeric matrices while maintaining consistent flowability during the extrusion process.

All designed formulations were developed to assess printability, thermal stability (TGA), and mechanical performance. Raw materials (PCL, HA, HAc, TCP, TCPc) and scaffold samples (SSs) of all formulations underwent spectroscopic characterisation by Fourier Transform Infrared (FTIR) and morphological and elemental analysis using a Scanning Electron Microscopy (SEM) combined with Energy Dispersive X-ray Spectroscopy (EDS) to analyse the materials further. Contact angle goniometry (CAG) analysis and cell viability studies were conducted specifically for PCL, PH10, PH20, PT10, and PT20. These formulations were selected based on the bioactive properties of HA and TCP.

#### 2.2.2. Fabrication Process of 3D-Printed Scaffolds

**Figure 1:**
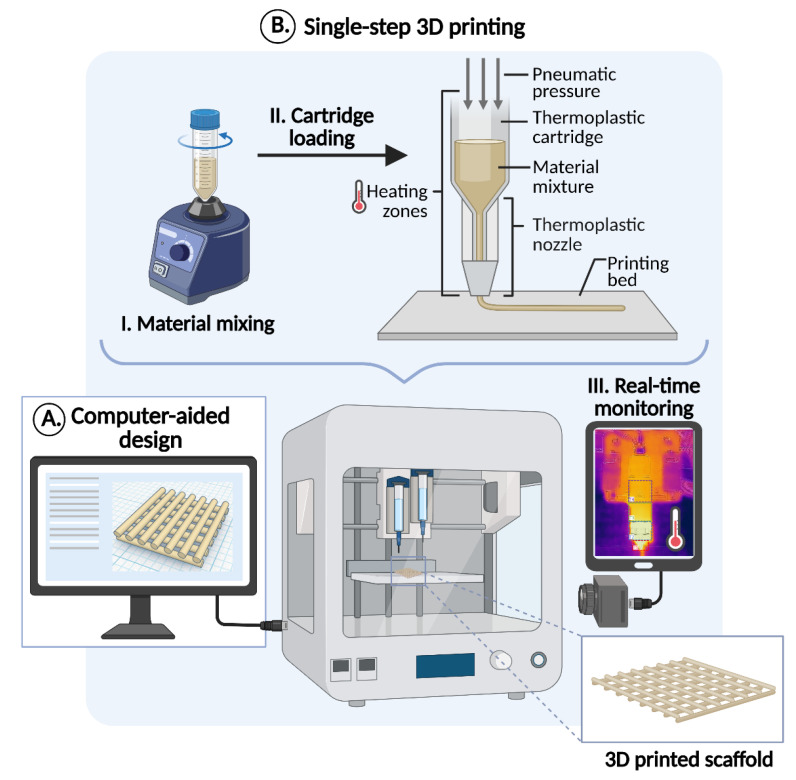
Illustration of the 3D printing process (created with BioRender.com (© 2024 BioRender)).

A single-step 3DP process was developed, utilising the melt-extrusion technique (Figure 1). Bilayer rectilinear scaffolds were designed with a raster orientation of 0°/90° and an internal square pattern design. This design was specifically developed to facilitate possible implantation on the external surface of bone tissue, serving as a supportive structure for bone fragments. The structural characteristics of the scaffold, particularly its square macroporosity, were intentionally chosen to enhance surgical handling by facilitating easier manipulation during implantation. The geometrical attributes of scaffolds ((a) square: width = length = 10.92 mm, height = 1.68 mm; (b) rectangular: length = 32.76 mm, width = 10.92 mm, height = 1.68 mm) were developed using Tinkercad^®^ (Autodesk Inc., CA, USA), following an optimised mathematical pattern, following a previous study of Dedeloudi et al. [30]. Implementing an optimised path is fundamental for achieving high printing precision, shape fidelity, and mechanical stability in the fabricated scaffold. The finalised designs were subsequently converted into Stereolithography (.stl) files and imported into the printer’s software (HeartOS ver. 1.8.2, Cellink, Göteborg, Sweden).

Initially, the powder-based physical mixture (PM) of the tested formulation was mixed (vortex, 5 min) and loaded into a 10 mL stainless steel syringe equipped with an 18G (0.84 mm) nozzle. The syringe was then fitted into a thermoplastic printhead (TP) and mounted onto a 3D Bio-X bioprinter (Cellink, Göteborg, Sweeden). The printing parameters were optimised using the printer’s software to assess printability. A constant extrusion temperature of 150 °C and a printing speed of 1 mm/s were maintained, while the applied pressure was varied between 75 and 90 kPa to evaluate the extrudability and deposition efficiency of the molten composites [16]. The extruded material was directly patterned into micro-dimensional rasters, avoiding the requirement for initial filament formation. Regarding system’s temperature control, the TP employs a closed-loop thermal regulation system that integrates internal heating elements, real-time temperature sensors, and Proportional Integral Derivative (PID) control algorithms, all governed through software monitoring, to ensure precise and consistent temperature maintenance for the accurate extrusion of thermoplastic biomaterials. Additionally, during the printing time, an IR-camera was placed at a 15 cm distance from the printing platform to monitor the system’s temperature in real time. Data acquisition and analysis were performed using the Vernier Thermal Analysis^®^ Plus app (Vernier Software & Technology, Beaverton, OR, USA), and temperature was verified by an external thermometer (±1 °C) [31].

#### 2.2.3. Morphological Characterisation

The dimensional and structural characteristics of the scaffold samples were inspected with optical microscopy and scanning electron microscopy (SEM), respectively.

A Leica Microsystems EZ4W optical microscope was employed to measure the raster width of the 3D-printed structures to assess shape fidelity (SF) relative to its theoretical dimensions (digital pattern), by calculating the % relative error and the printability index (Pr), as determined by Equation (1) [15].

(1) Printability index = 1−experimental dimension − digital dimensiondigital dimension

where 0 < Pr ≤ 1, the experimental dimension corresponds to the actual raster diameter of the printed scaffold, while the digital dimension to the CAD values. Pr values closer to 1 indicate higher accuracy and conformity between the printed scaffold and the digital design.

The surface morphology, topology, and pore formation of the ceramic microparticles and scaffolds were examined with SEM (SNE Alpha, SEC Co., Ltd., Seoul, Republic of Korea), operated under a vacuum at 10–20 kV and 0.5–5k magnification. Elemental composition was determined through EDS using a Bruker Nano GmbH system (Berlin, Germany) and analysed with Bruker ESPRIT Compact software, ver. 2.1 (Bruker Nano GmbH, Berlin, Germany).

#### 2.2.4. Fourier Transform Infrared Spectroscopy

FTIR analysis was conducted on all raw materials and scaffold samples to assess the uniformity and consistency of each ceramic in the polymeric matrix, identify the components, and investigate possible chemical interactions between the materials. A Nicolet™ iS50FTIR Spectrometer (ThermoFisher Scientific Inc., Waltham, MA, USA) equipped with attenuated total reflectance (ATR) was used for the analysis. All samples were tested in triplicate, with a 4 cm^−1^ resolution and 64 scans, over a spectral range from 4000 cm^−1^ to 400 cm^−1^.

#### 2.2.5. Thermogravimetric Analysis

The thermal stability and the residual weight (%) of all extruded scaffolds were evaluated using a Q500 TGA (TA Instruments, New Castle, DE, USA). Samples (*n* = 3, 5–10 mg) were placed in an open aluminium pan and subjected to a thermal degradation process from 20 °C to 500 °C, applying a 20 °C/min heating rate under a nitrogen flow rate of 40 mL/min. The residual weight (%) confirmed the incorporated wt% ceramic in the 3D-printed structures.

#### 2.2.6. Mechanical Analysis of Tensile Strength

The tensile strength of all extruded scaffolds was evaluated until failure to determine their elastic behaviour. Rectangle samples (*n* = 5) were tested via a MultiTest-dV 2.5 (500 N load capacity) (Mecmesin, Horsham, UK), at a 20 mm vertical position and a 5 mm/s speed. Young’s Modulus (E), elastic strain limit (ε_elastic_), Ultimate Tensile Strength (UTS), strain at UTS (ε_UTS_), and strain at failure (ε_f_) were calculated from the stress vs. strain curve [32].

#### 2.2.7. Water Contact Angle

The surface wettability of scaffold samples was assessed using a Theta Flow Optical Tensiometer (Biolin Scientific, Västra Frölunda, Sweden) employing the sessile drop technique. A 1 μL droplet of deionised water was precisely deposited at the centre of each raster using an automated pipette (incorporated probe). The static contact angle (CA) was evaluated from the initial image captured 1 s post-droplet deposition. Measurements were performed in triplicate at different locations on the upper surface of each scaffold, and data acquisition was carried out using OneAttension software, with an accuracy of ±0.1°.

#### 2.2.8. Cell Studies

Human lung fibroblasts (PromoCell) were used to examine their adherence and biocompatibility with the scaffold. Cells between passage 3 and passage 5 were selected for the experiments. Scaffolds were initially sterilised in 70% ethanol, washed with PBS and exposed to UV light for 20 min, subsequently placed in a 24-well plate for the assay. Cells were seeded at 20,000 cells density in DMEM (ThermoFisher Scientific) supplemented with 10% Fetal Bovine Serum (ThermoFisher Scientific). Fibroblasts were cultured in absence of scaffolds as control. PCL scaffold was used as a toxicity control. Media was changed after 48–72 h. Cells were imaged at day 2 and day 5 in culture. Phase contrast imaging was used to assess cell growth, morphology and viability. After 5 days in culture, cell fixation was achieved by using 4% Formaldehyde (ThermoFisher Scientific) for 15 min at room temperature. After fixation, DAPI (ThermoFisher Scientific) staining was performed using a 1:1000 concentration in 1X PBS (Sigma Aldrich, Stenheim, Germany) for 15 min at room temperature to visualise cell nuclei and to assess cell viability on scaffolds. Images of scaffolds were acquired using the Leica DM5500 microscope (Leica Microsystems GmbH, Wetzlar, Germany). This study was constituted as a qualitative proof-of-concept assessment of the model, and the experiment was conducted once (*n* = 3; 3 × wells per condition).

#### 2.2.9. Statistical Analysis

Mechanical analysis results were statistically evaluated using one-way analysis of variance (ANOVA), followed by Tukey’s post hoc test to assess group differences (GraphPad Prism^®^ software ver.9; GraphPad Software Inc., San Diego, CA, USA). A confidence level of 95% was applied, with a significance threshold set at *p* < 0.05. All data are expressed as the average (Avg.) ± standard deviation (S.D.), with at least triplicates for each group.

## 3. Results and Discussion

### 3.1. Printability Studies

Printability studies were performed to fabricate polymeric-ceramic bilayer rectilinear scaffolds. Printing parameters such as temperature, pressure, and speed were examined to assess their impact on shape fidelity, surface morphology, and mechanical integrity. The values were adjusted according to Dedeloudi et al. [30], following the mathematical model of % infill and achieving similarity on 3D structure. Temperature settings followed the temperature zones of the printhead during temperature adjustments. The loaded PMs were heated up to 150 °C, higher than the polymer’s melting point (T_m(PCL)_ ≈ 65 °C) [30], assuring efficient melting behaviour, subsequently creating a viscous environment for the suspended in the matrix ceramic. Moreover, pressure values were examined prior to printing process to assess the extrudability of the semi-molten PM. The ceramic particle size (d_50_) was significantly smaller than the nozzle diameter (d_50_/d_nozzle_ ≈ 1/170), ensuring extrusion efficiency and positively impacting overall printability. Therefore, pressure values for all polymeric-ceramic composites exhibited a slight increase when using higher ceramic wt%, following a directly proportional trend (Table 2), likely due to the increase in compactness of the solid ceramic microparticles in the polymeric melt. Additionally printing speed was maintained at 1 mm/s, ensuring uniform particle distribution at the extrusion edge; hence was determined to be optimal for these formulations. The combination of controlled pneumatic pressure and speed contributed on the homogeneous dispersion of ceramic particles within the polymeric matrix during printing, resulting in a stable and well-defined 3D structure [33].

**Figure 2:**
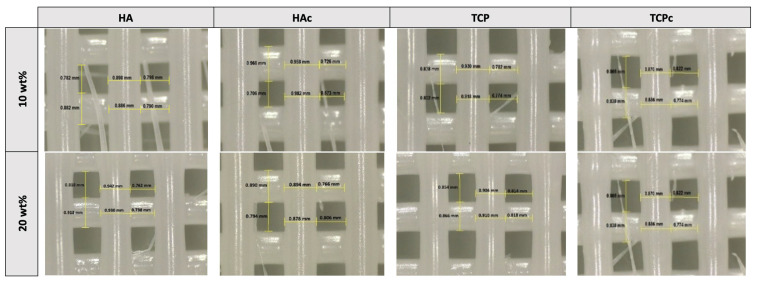
Optical microscope images showing raster dimensions of bilayer 3D-printed polymeric-ceramic composite scaffolds. The wt% indicates the proportion of the incorporated ceramic into PCL formulations.

All PCL–ceramic formulations showed a negative value (PTc20, min: −2.08%; PT10, max: −10.60%) for the raster width, denoting material expansion after deposition. The printability index ranged from 0.89 to 0.98, indicating excellent shape fidelity across all scaffold structures and demonstrating a high level of printing precision (Figure 2).

### 3.2. Microstructural Characteristics of the 3D-Printed Scaffolds

**Figure 3:**
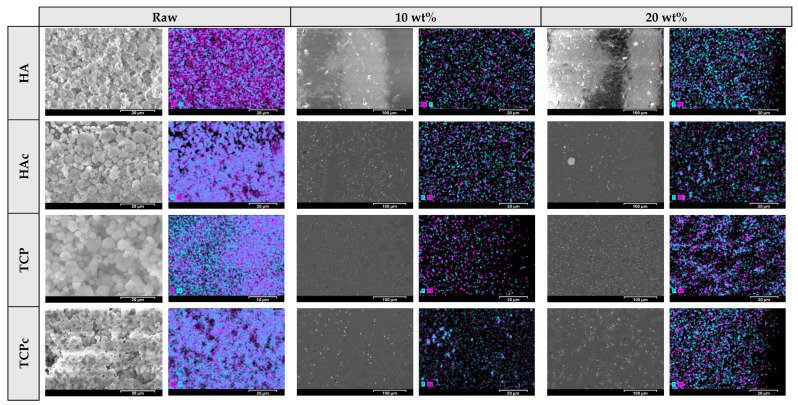
SEM-EDS analysis of ceramic materials and PCL-ceramic composite samples. The left image illustrates the surface morphology; where HA’s surface characteristics were investigated in the relevant study of Dedeloudi et al. [16]. The right image presents the elemental mapping of all ceramics and the developed PCL–ceramic composite samples. The wt% indicates the proportion of the incorporated ceramic into PCL formulations.

SEM-EDS was conducted to assess the architectural properties and elemental consistency of all ceramic microparticles and the 3D-printed scaffolds (Figure 3). The HA microparticles exhibited spherical and smooth morphologies, while HAc appeared to possess curved surfaces and cavities. On the raster surface of PH scaffolds, distinct, bright, spherical structures representing HA were identified, uniformly blended with the polymer [34]. It was observed that the quantity of ceramic microparticles became more compact as their wt% increased. A similar image was obtained for PHc scaffolds, with well-defined small microparticles dispersed throughout the PCL matrix. These microparticles appeared to be evenly distributed in PHc10 scaffolds, with their prevalence increasing proportionally to the HAc concentration. Conversely, a noticeable aggregation of HAc was detected in specific regions on PHc20 scaffolds. This localised particle compaction was attributed to the low surface energy of the ceramic, which probably induced attractive interparticle forces, leading to cluster formation [35].

TCP microparticles had a smooth surface and spherical shape, in contrast to TCPc, which appeared to have an irregular morphology, forming agglomerates. PT scaffolds showed an efficient distribution of TCP throughout the PCL surface, while its appearance was directly relative to the wt% of the ceramic. The efficiency of particle distribution was associated with the lower MW, in contrast to HA, and its absence of hydroxyl groups, therefore avoiding possible interactions with the hydrophobic matrix. A similar image regarding the particle distribution was acquired for all PTc extruded scaffolds. TCPc’s nonspherical structural characteristics reduced the likelihood of particle agglomeration, improving its flowability and, subsequently, the 3DP process and their dispersion along the matrix.

All Ca:P proportions (i.e., distinct for the ceramic chemical structure) detected on the scaffold surface were equal to evaluated ratios of the relevant ceramic microparticles (Figure S1), determining a precise consistency of the ceramics into the polymeric matrix. Additionally, elemental maps showed adequate distribution of Ca and P on the scaffolds’ surfaces, which is comparable to SEM images. The similarity of these captures confirmed the homogeneity of both PCL and ceramics into the final extrudate.

### 3.3. FTIR Characterisation

**Figure 4:**
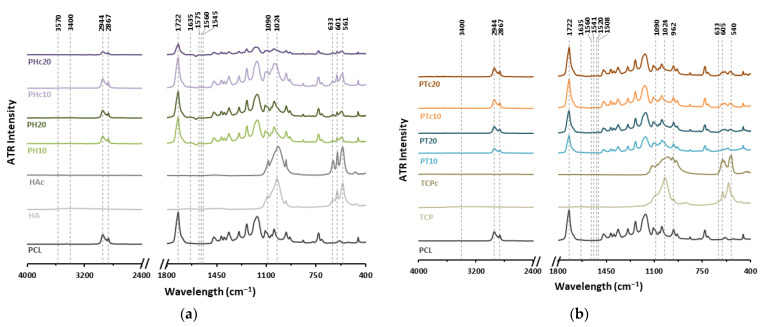
ATR spectra illustrating significant absorbance bands of all (**a**) PH and PHc, and (**b**) PT and PTc scaffolds in comparison to their incorporated ceramic and PCL.

FTIR analysis revealed characteristic peaks corresponding to all raw materials, confirming their presence and indicating efficient ceramic dispersion within the extruded samples (Figure 4). Owing to the different physical states of the ceramics, common peaks appeared at 1090 cm^−1^ and 1024 cm^−1^, corresponding to the asymmetric stretching mode (*v*3) of the phosphate group (${\mathrm{PO}}_{4}^{3-}$). These peaks appeared slightly broadened in both HAc and TCPc, attributed to heat-induced water loss (e.g., hydroxyl groups), which affected phosphate bonding, leading to microstrain and lattice defects. Moreover, a broad peak in the 3200–3400 cm^−1^ range was present in both HA and TCP, indicative of absorbed water molecules on their surface, contributing to hydrogen bonding, which was not detectable in calcined ceramics. The stretching vibration at 3570 cm^−1^, associated with structural hydroxyl groups (O-H), was evident in HA and at a lower intensity in HAc [36], whereas it was not observed in TCP and TCPc.

In the fingerprint region, bending modes (*v*4) of the ${\mathrm{PO}}_{4}^{3-}$ were observed at 561 cm^−1^ and 601 cm^−1^ for both PH and PHc formulations, along with a hydroxyl stretching vibration at 633 cm^−1^ [37]. In contrast, TCP and TCPc exhibited these phosphate bending absorptions at 540 cm^−1^ and 605 cm^−1^, while the hydroxyl vibration was absent due to calcination process. Moreover, a strong sharp peak at 962 cm^−1^, representative for the symmetric P-O stretching (*v*1) of ${\mathrm{PO}}_{4}^{3-}$, determined the *β*-crystalline nature of TCP [38]. However, this peak overlapped with the ether (C-O-C) stretching of PCL, making it hardly visible in composite formulations.

Between 1500 cm^−1^ and 1650 cm^−1^, weak and small peaks were observed in all composite formulations. Peaks at 1545, 1560, and 1570 cm^−1^ indicated possible stretching vibrations of the carboxylate (-COO^−^) group of the PCL, interacting with the ceramics’ calcium (Ca^2+^) in PH and PHc formulations. Similar peaks were detected for PT and PTc formulations at 1508, 1520, 1541, and 1560 cm^−1^. Additionally, a peak at 1635 cm^−1^, present in all scaffolds, was attributed to absorbed water (H-O-H bending) on ceramics’ surfaces or to a potential shift in the carbonyl (C=O) stretching of PCL from 1722 cm^−1^, due to its partial hydrolysis throughout the heat-extrusion process.

Overall, PCL demonstrated good compatibility within ceramic mixtures, maintaining its structural integrity. Characteristic peaks of symmetric and asymmetric C-H stretching of CH_2_ groups were observed at 2867 cm^−1^ and 2944 cm^−1^, respectively, confirming the retention of its polymer backbone features [30].

### 3.4. Thermal Degradation Analysis

**Figure 5:**
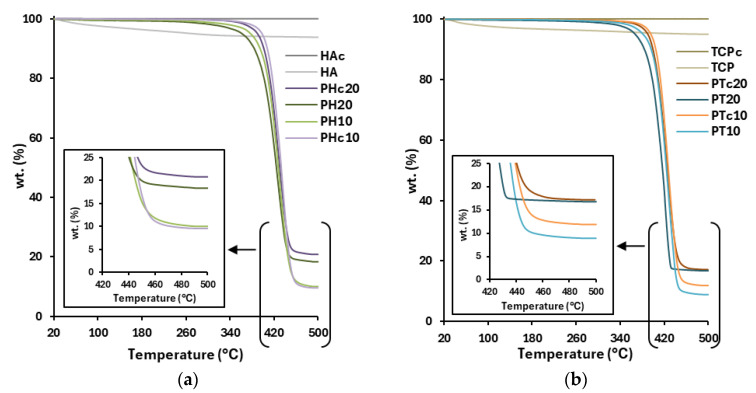
TGA thermograms of (**a**) HA, HAc, and all PH and PHc 3D-printed scaffolds and (**b**) TCP, TCPc and all PT and PTc 3D-printed scaffolds.

Thermogravimetric (TGA) analysis was conducted on all PCL–ceramic samples to evaluate their thermal degradation profile and determine the wt% of ceramics incorporated into the extruded matrixes (Figure 5). All measurements were compared to blank PCL scaffolds, selecting 500 °C as the end temperature, representative of PCL’s absolute mass loss [39]. Both raw HA and TCP showed comparable % weight loss at 500 °C (HA: 93.73 ± 0.13%, TCP: 94.88 ± 0.17%). In contrast, their calcined counterparts (i.e., HAc and TCPc) did not undergo any thermal degradation, indicating enhanced thermal stability under heat-induced processes.

PHc20 scaffolds demonstrated a higher ceramic content than PH20 (PH20: 16.29 ± 0.92%; PHc20: 21.33 ± 0.52%), suggesting improved ceramic retention in the calcined variant. It is assumed that HAc’s coarsened surface facilitated enhanced PCL infiltration into its microcavities, thereby promoting its efficient suspension within the molten polymeric matrix [35]. Regarding PT and PTc scaffolds, the concentration of calcined TCP appeared generally higher across all PT formulations (Table 3). The lower surface energy of both calcined ceramics resulted in a more compact blend with the lipophilic PCL, reducing particle mobility and promoting their greater inclusion within the polymer matrix. The observed reduction in ceramic mass (wt%) occurred during the printing process, likely because of the pneumatic pressure applied during extrusion. Consequently, calcining enabled the control of the powder’s properties, thereby optimising the characteristics of the final scaffold.

### 3.5. Mechanical Analysis

The mechanical behaviour of all extruded composite scaffolds was evaluated in terms of their ductility and brittleness during the tensile testing process. Key parameters related to stiffness such as Young’s modulus (E) and elastic strain limit (ε_elastic_), as well as to those related to plasticity and load capacity, including ultimate tensile strength (UTS) and strain at UTS (ε_UTS_), and ductility (strain at failure, ε_f_), highlighted significant critical differences in the tensile strength properties of the formulations (Table 4).

**Figure 6:**
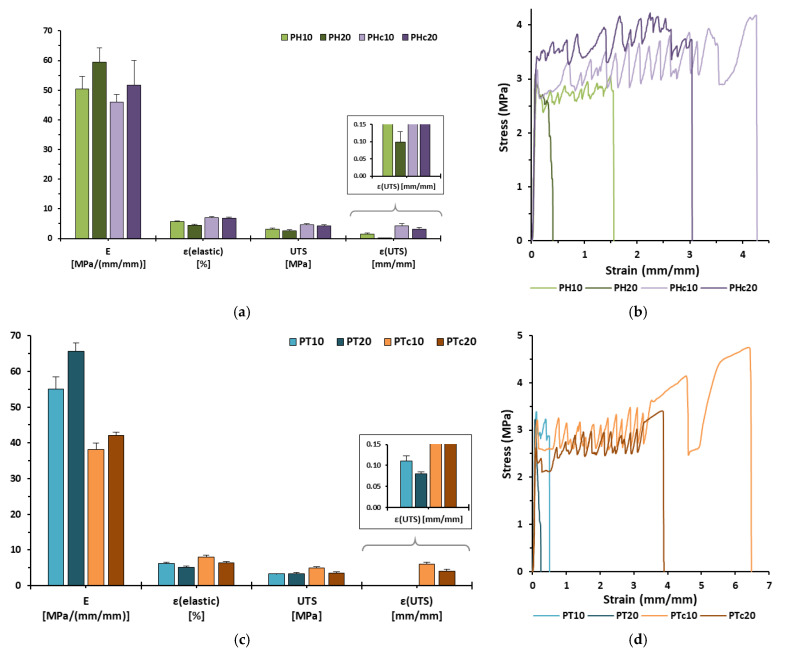
(**a**–**c**) Bar charts illustrating Young’s Modulus (E), elastic strain limit (ε_elastic_), Ultimate Tensile Strength (UTS), and strain at UTS (ε_UTS_) (Avg. ± S.D.; *n* = 5), and (**b**–**d**) representative stress–strain curves of all 3D-printed scaffolds, respectively.

Each formulation consisted of 10 wt% ceramic contributed to a less stiff and more ductile mechanical profile compared to the relative 20 wt% ceramic formulation (Figure 6a–c). These results demonstrated that adding ceramics played a pivotal role in enhancing the stiffness of the scaffolds while simultaneously reducing their elastic characteristics [40]. Moreover, scaffolds containing calcined ceramics (both HAc and TCPc loaded scaffolds) exhibited improved elasticity and ductility compared to their non-calcined counterparts [41,42]. Specifically, the PTc10 was characterised as the least stiff structure (E: 38.17 ± 1.76 MPa) and exhibited high elasticity (ε_elastic_: 8.01 ± 0.56%), plasticity (UTS: 4.92 ± 0.41 MPa; ε_UTS_: 6.10 ± 0.43) and ductility (ε_f_: 664.07 ± 32.82%) in comparison to the other formulations. This behaviour is attributed to the lower surface energy and higher porosity of the calcined ceramic microparticles, which promoted a more efficient integration with the molten polymer matrix. In contrast, both PH20 and PT20 were identified as the stiffest structures, showing similar mechanical values; hence, they were characterised by low elasticity while achieving the highest brittleness (ε_UTS_, PH20: 0.10 ± 0.03, PT20: 0.08 ± 0.01). The drastic decrease in ductility is possibly sequential to the more compact crystalline structure and denser atomic packing of the non-calcined ceramics, which hindered the scaffold’s ability to deform plastically. All in all, stress–strain curves revealed the vertical stretch of 3D-printed rasters, with reproducible curve patterns for each formulation that were closely related to the ductility characteristics of each respective formulation (Figure 6b–d) [30].

The bilayer pattern was strategically created to enable potential implantation on the outer surface of bone tissue, serving as a supportive structure for fractured bone segments. A study of Popowics et al. [43] which investigated the mechanical attributes of the periosteum (i.e., a bilayer connective tissue membrane that surrounds the external surface of bones, offering structural support and facilitating bone healing [1]) of the mandibular body in pigs revealed tensile strength characteristics (E = 63.0 ± 25.4 MPa; UTS = 8.2 ± 4.1 MPa) comparable to those of the developed 3D-printed scaffolds. Therefore, the proposed formulations hold promise as artificially fabricated biomaterial-based tissue for bone segmental support and restoration.

### 3.6. Cell Attachment and Proliferation Studies

**Figure 7:**
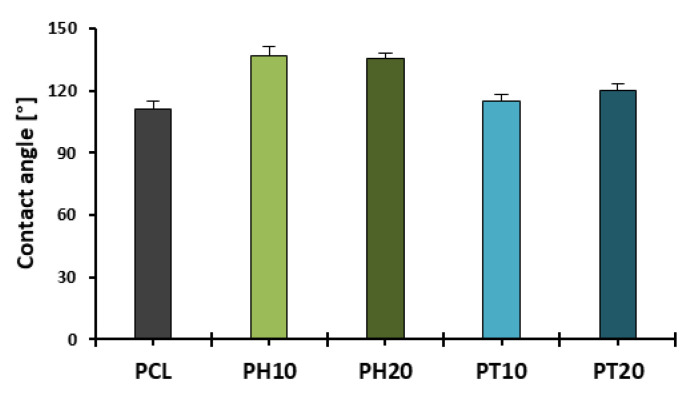
Contact angle measurements of 3D-printed scaffolds following droplet deposition (t = 1 s) (Avg. ± S.D.; *n* = 3).

**Figure 8:**
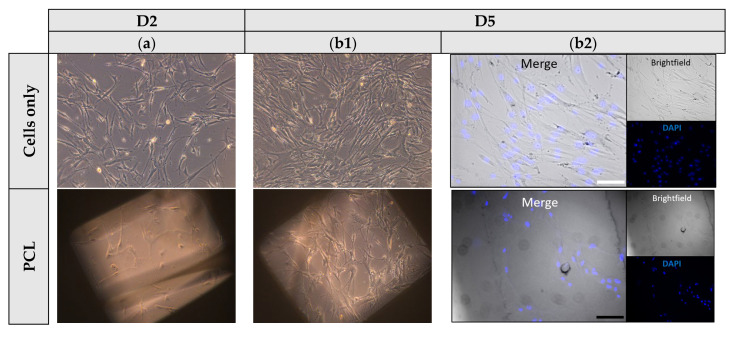

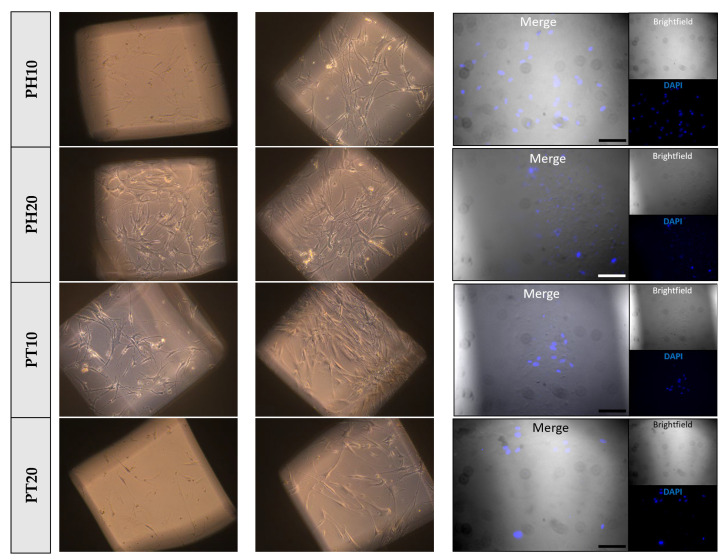
Representative brightfield images depicting fibroblasts at (**a**,**b1**) days 2 and 5 (D2,D5) of culture, and (**b2**) DAPI (blue) stained images of cells cultured on all PH and PT 3D printed scaffolds (20X; scalebar: 100 μm).

Cells were seeded on PCL, PH, and PT scaffolds, and their viability, morphology, and growth were evaluated over the testing period. A cells-only group was used as positive control, to confirm cell growth and cell viability. Among the scaffolds, higher efficiency in cell attachment and growth was shown to be on PCL scaffolds, as observed on day 5. Contrarily, PT20 exhibited limited cell attachment and growth compared to the other formulations. The latter may be due to its stiffer structure and the micro-roughness of the crystalline TCP particles. Moreover, the lipophilic nature of the scaffolds (CA > 90°) (Figure 7 and Figure S2), along with the round geometric shape of the rasters, did not enable the complete spreading of cells across the surface, potentially due to gravitational forces affecting distribution (Figure 8) [39].

The present study evaluated the capacity of fibroblasts to adhere and proliferate on PCL and PCL-bioceramic composite scaffolds, suggesting that final compositions are defined as cytocompatible and non-toxic platforms. These findings are consistent with previous studies highlighting the suitability of PCL-based biomaterials for cell adhesion and growth [44], while the incorporation of bioceramic phases may further enhance the biological performance of the scaffold [45]. Given that fibroblasts, as mesenchymal-derived cells, possess the intrinsic capacity to differentiate and contribute to tissue remodelling, their favourable response to these scaffolds highlights their potential utility in regenerative applications [46]. Importantly, the incorporation of these biomaterial-based scaffolds at the muscle-bone interface could create new perspectives for investigating mechanotransducive processes, which remain a critical aspect of musculoskeletal regeneration. Collectively, these preliminary results support continued evaluation of PCL-based composites in the context of in vitro tissue modelling, as well as toxicological and pharmaceutical testing.

## 4. Conclusions

This study highlighted the application of a single-step melt-extrusion 3DP process for developing biomaterial-based scaffolds intended for the restoration of segmental bone defects. Three-dimensional technique was employed successfully for the fabrication of geometrically precise bilayer rectilinear implants, consisting of PCL and HA and TCP bioceramics, in both their bioactive and calcined forms. The chemical composition of all formulations exhibited compatibility between the polymer and the ceramics. Degradation temperature confirmed the integrity, stability, and adequate ceramic consistency within the polymeric scaffolds, and morphological surface assessment and elemental analysis further indicated a homogenous dispersion of ceramics into 3D-printed structures. Moreover, mechanical testing revealed a higher brittleness in bioceramic-based scaffolds, while calcined ceramics performed a more ductile profile, showcasing enhanced plasticity. All materials exhibited cytocompatibility and supported cell proliferation, underscoring the need for further investigations into long-term cellular responses, differentiation capacity, and functional integration within relevant microenvironments. Collectively, these findings underline manufacturing steps for developing tailor-made scaffolds with varying elastic profiles for potential post-surgical bone tissue restoration.

## Figures and Tables

**Table 1:** Composition of applied formulations.

Formulation ^a,b^	Composition (wt%)				
	PCL	HA	HAc	*β*-TCP	*β*-TCPc
PH10	90	10	-	-	-
PH20	80	20	-	-	-
PHc10	90	-	10	-	-
PHc20	80	-	20	-	-
PT10	90	-	-	10	-
PT20	80	-	-	20	-
PTc10	90	-	-	-	10
PTc20	80	-	-	-	20

^a^ PMs were developed for all composite formulations. ^b^ P: PCL; H: HA; Hc: HAc; T: TCP; Tc: TCPc, and the number following the letters re resents the wt% of the respective ceramic.

**Table 2:** Printability evaluation of polymeric-ceramic composite scaffolds. Dimensional characteristics and printability index (Pr) denote their shape fidelity.

Formulation	Printing Pressure (kPa)	Raster Width		Pr
		(mm)	Relative Error (%)	
PH10	75	0.89 ± 0.04	−5.79	0.93
PH20	90	0.88 ± 0.04	−4.38	0.95
PHc10	80	0.90 ± 0.02	−7.06	0.93
PHc20	90	0.87 ± 0.03	−3.02	0.97
PT10	70	0.93 ± 0.04	−10.60	0.89
PT20	85	0.90 ± 0.04	−6.61	0.91
PTc10	75	0.88 ± 0.02	−4.56	0.95
PTc20	85	0.86 ± 0.02	−2.08	0.98

**Table 3:** Thermal degradation properties of all raw materials and scaffold samples (Avg. ± S.D.; n = 3).

	Formulations	Residual wt (%) (T = 500 °C)
Raw Materials	HA	93.73 ± 0.13
	HAc	100.00
	TCP	94.88 ± 0.17
	TCPc	100.00
Scaffold Samples *	PH10	8.82 ± 0.59
	PH20	16.29 ± 0.92
	PHc10	9.56 ± 0.01
	PHc20	21.33 ± 0.52
	PT10	8.64 ± 0.19
	PT20	15.89 ± 0.03
	PTc10	12.02 ± 0.14
	PTc20	17.08 ± 0.11

* PH and PT formulations represent the normalised residual wt (%), based on the weight loss of the incorporated ceramic.

**Table 4:** Mechanical attributes (E, ε_elastic_, UTS, ε_UTS_ and ε_f_) of all 3D-printed scaffolds (Avg. ± S.D.; n = 5). The statistical significance is reported in Table S2.

Formulation	E [MPa/(mm/mm)]	ε_elastic_ [%]	UTS [MPa]	ε_UTS_ [mm/mm]	ε_f_ [%]
PH10	50.32 ± 4.26	5.69 ± 0.28	3.16 ± 0.29	1.45 ± 0.33	153.58 ± 40.32
PH20	59.46 ± 4.73	4.50 ± 0.38	2.67 ± 0.25	0.10 ± 0.03	64.06 ± 21.11
PHc10	46.03 ± 2.45	7.07 ± 0.34	4.58 ± 0.34	4.32 ± 0.61	489.00 ±60.01
PHc20	51.69 ± 8.34	6.89 ± 0.37	4.21 ± 0.37	3.12 ± 0.62	350.20 ± 32.57
PT10	55.09 ± 3.41	6.12 ±0.41	3.36 ± 0.03	0.11 ± 0.01	64.58 ± 11.95
PT20	65.66 ± 2.33	5.19 ± 0.19	3.41 ± 0.24	0.08 ± 0.01	28.14 ± 2.62
PTc10	38.17 ± 1.76	8.01 ± 0.56	4.92 ± 0.41	6.10 ± 0.43	664.07 ± 32.82
PTc20	42.06 ± 0.90	6.43 ± 0.25	3.50 ± 0.38	4.04 ± 0.63	410.91 ± 65.32

## Data Availability

The data supporting the findings of this study are available within the article. Additional data are available from the corresponding author upon reasonable request.

## Supplementary Materials

The following supporting information can be downloaded at: https://www.mdpi.com/article/10.3390/jfb16100358/s1, Figure S1: EDS spectrum depicting the Ca:P ratios for raw ceramics and their PCL-based 3D extruded scaffolds. The wt% consistency indicates the proportion of the incorporated ceramic into PCL formulations; Figure S2: Representative front-lit illumination setup for contact angle (CA) measurements of droplets on a scaffold’s raster-patterned surface. Table S1: Physical characteristics of all ceramics microparticles, as provided in the manufacturer’s (FLUIDINOVA, S.A.) certification of analysis document; Table S2: Contact angle goniometry testing for 3D-printed scaffold samples (Avg. ± SD; n = 3); Table S3: One-way ANOVA results for (a) Young’s modulus (E), (b) Ultimate Tensile Strength (UTS), (c) strain at UTS (ε_UTS_), (d) elastic strain limit (ε_elastic_), (e) strain at failure (ε_f_) for all 3D-printed scaffolds.

## Abbreviations

The following abbreviations are used in this manuscript:

3DP	3D printing
AM	additive manufacturing
ANOVA	One-way Analysis of Variance
ATR	Attenuated Total Reflectance
Avg.	average
BTR	bone tissue regeneration
CA	contact angle
CAD	Computer-Aided Design
CAG	Contact Angle Goniometry
DAPI	4′,6-diamidino-2-phenylindole
DIW	Direct Ink Writing
DMEM	Dulbecco’s Modified Eagle’s Medium
E	Young’s modulus
ECM	Extracellular Matrix
EDS	Energy-Dispersive X-ray
EM	emerging manufacturing
FBS	Fetal Bovine Serum
FFF	Fused Filament Fabrication
FTIR	Fourier-transform infrared spectroscopy
GelMA	gelatin methacryloyl
HA	hydroxyapatite
HAc	calcined hydroxyapatite
HPF	Primary Human Pulmonary Fibroblasts
HUVEC	Human Umbilical Vein Endothelial Cells
IR	Infra-red
MSCs	Mesenchymal Stem Cells
MW	Molecular Weight
PBS	Phosphate-Buffered Saline
PCL	polycaprolactone
PLGA	polylactic(L-lactic-co-glycolic acid)
PM	Physical Mixture
PID	Proportional Integral Derivative
PUs	polyurethane
S.D.	Standard Deviation
SEM	Scanning Electron Microscope
SF	Shape Fidelity
SSs	Scaffold Samples
TCP	tricalcium phosphate
TCPc	calcined tricalcium phosphate
T_deg_	degradation temperature
TE	Tissue Engineering
TGA	Thermogravimetric Analysis
T_m_	melting temperature
TP	Thermoplastic Printhead
UTS	Ultimate Tensile Strength
ε_elastic_	elastic strain limit
ε_f_	strain at failure
ε_UTS_	strain at UTS
