# Supplementary material for: Development of Bioceramic Bone-Inspired Scaffolds Through Single-Step Melt-Extrusion 3D Printing for Segmental Defect Treatment
Source: J Funct Biomater. 2025 Sep 23;16(10):358. doi: 10.3390/jfb16100358 (PMC12565026; doi:10.3390/jfb16100358)
Supplement: Supplementary file 1 [file jfb-16-00358-s001.zip › jfb-3828593-supplementary.pdf]

Journal of
Functional Biomaterials

MDPI

Article

Development of bioceramic bone-inspired scaffolds through single-step melt-extrusion 3D printing for segmental defect treatment

Aikaterini Dedeloudi[1], Pietro Maria Bertelli[2], Laura Martinez-Marcos[3], Thomas Quinten[3], Imre Lengyel[2], Sune K. Andersen[3], Dimitrios A. Lamprou[1]*

[1] School of Pharmacy, Queen's University Belfast, 97 Lisburn Road, Belfast BT9 7BL, UK; adedeloudi01@qub.ac.uk (A.D.)
[2] Wellcome-Wolfson Institute for Experimental Medicine, School of Medicine, Dentistry and Biomedical Sciences, Queen's University Belfast, 97 Lisburn Road, Belfast BT9 7BL, UK; p.bertelli@qub.ac.uk (P.M.B); i.lengyel@qub.ac.uk (I.L.)
[3] Johnson & Johnson Innovative Medicine, Oral Solids Development, Research & Development, Turnhoutseweg 30, 2340 Beerse, Belgium; LMart122@ITS.JNJ.com (L.M.M); tquinte@ITS.JNJ.com (T.Q.); SAnder83@its.jnj.com (S.K.A.)
* Correspondence: d.lamprou@qub.ac.uk (D.A.L.)

. Supplementary Materials

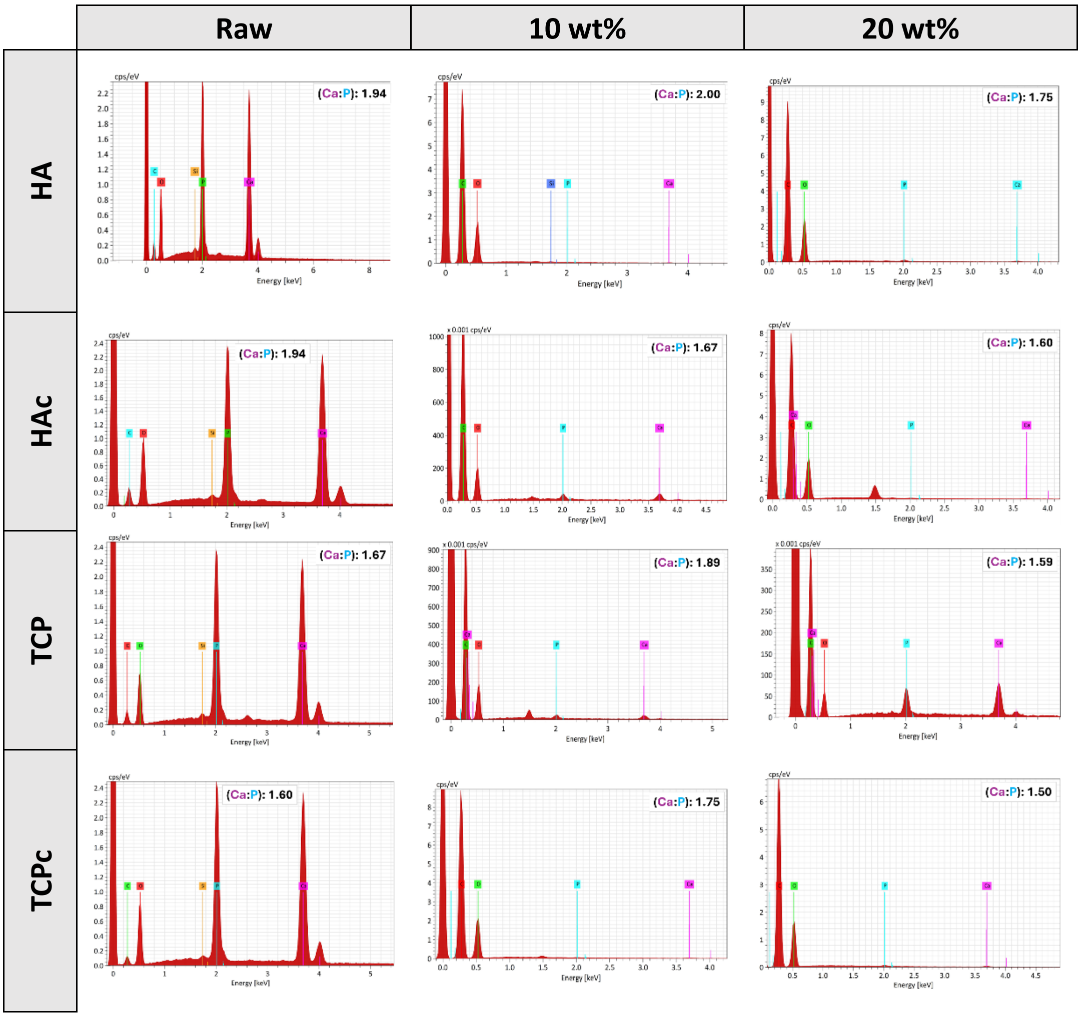

Figure S1. EDS spectrum depicting the Ca:P ratios for raw ceramics and their PCL-based 3D extruded scaffolds. The wt% consistency indicates the proportion of the incorporated ceramic into PCL formulations.

https://doi.org/10.3390/xxxxx

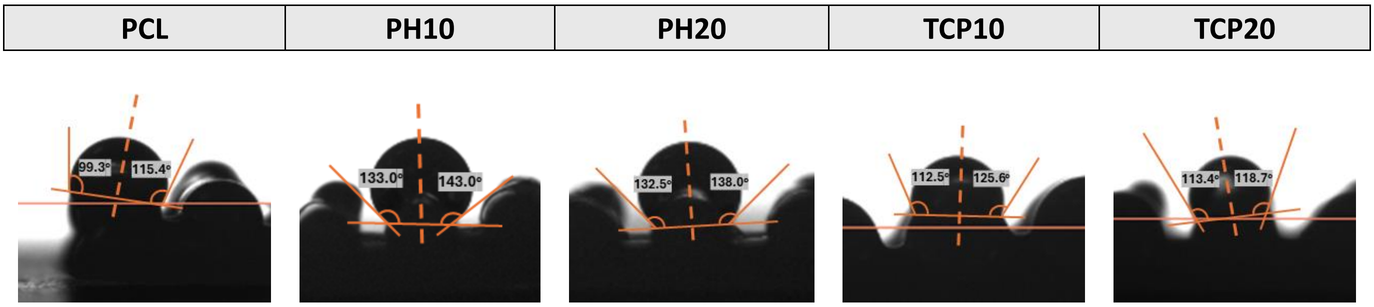

Figure S2. Representative front-lit illumination setup for contact angle (CA) measurements of droplets on a scaffold's raster-patterned surface.

Table S1. Physical characteristics of all ceramics microparticles, as provided in the manufacturer's (FLUIDINOVA, S.A.) certification of analysis document.

Material	Surface Area (m^2/g)	T_m (°C)	Water solubility (g/L, at 20°C)
**Hydroxyapatite** **$Ca_{10}(PO_4)_6(OH)_2$** **(nanoXIM·HAp202)**	>80 (high)	1670	0.00657
**Calcined hydroxyapatite** **$Ca_{10}(PO_4)_6(OH)_2$** **(nanoXIM·HAp602)**	1-10 (low)	1670	0.00657
**β-tricalcium phosphate** **$Ca_3(PO_4)_2$** **(nanoXIM·TCP200)**	>80 (high)	1670	0.0077
**Calcined β-tricalcium phosphate** **$Ca_3(PO_4)_2$** **(nanoXIM·TCP600)**	low	1670	0.0077

Table S2. Contact angle goniometry testing for 3D printed scaffold samples (Avg. ± SD; n = 3).

Formulation	CA mean [°]
**PCL**	111.2 ± 3.8
**PH10**	136.9 ± 4.6
**PH20**	135.3 ± 2.8
**PT10**	115.0 ± 3.1
**PT20**	120.3 ± 3.2

Table S3. One-way ANOVA results for (**a**) Young's modulus (E), (**b**) Ultimate Tensile Strength (UTS), (**c**) strain at UTS (ε_{UTS}), (**d**) elastic strain limit ($\varepsilon_{elastic}$), (**e**) strain at failure (ε_f) for all 3D printed scaffolds.

(**a**)

Tukey's multiple comparisons test	Mean Diff.	95.00% CI of diff.	Below threshold?	Summary	Adjusted P Value
PH10 vs. PH20	-9.138	-18.05 to -0.2253	Yes	*	0.0435
PH10 vs. PHc10	4.283	-4.630 to 13.20	No	ns	0.5318
PH10 vs. PHc20	-1.376	-10.29 to 7.537	No	ns	0.9703
PH20 vs. PHc10	13.42	4.509 to 22.33	Yes	**	0.0027
PH20 vs. PHc20	7.762	-1.151 to 16.67	No	ns	0.0996
PHc10 vs. PHc20	-5.660	-14.57 to 3.253	No	ns	0.3018
PT10 vs. PT20	-10.57	-14.37 to -6.762	Yes	****	<0.0001
PT10 vs. PTc10	16.91	13.11 to 20.72	Yes	****	<0.0001
PT10 vs. PTc20	13.02	9.219 to 16.83	Yes	****	<0.0001
PT20 vs. PTc10	27.48	23.67 to 31.28	Yes	****	<0.0001
PT20 vs. PTc20	23.59	19.79 to 27.40	Yes	****	<0.0001
PTc10 vs. PTc20	-3.888	-7.693 to -0.08338	Yes	*	0.0443

(**b**)

Tukey's multiple comparisons test	Mean Diff.	95.00% CI of diff.	Below threshold?	Summary	Adjusted P Value
PH10 vs. PH20	0.4934	-0.01675 to 1.004	No	ns	0.0598
PH10 vs. PHc10	-1.201	-1.711 to -0.6907	Yes	****	<0.0001
PH10 vs. PHc20	-1.049	-1.559 to -0.5385	Yes	***	0.0001
PH20 vs. PHc10	-1.694	-2.204 to -1.184	Yes	****	<0.0001
PH20 vs. PHc20	-1.542	-2.052 to -1.032	Yes	****	<0.0001
PHc10 vs. PHc20	0.1522	-0.3579 to 0.6623	No	ns	0.8282
PT10 vs. PT20	-0.05420	-0.6486 to 0.5402	No	ns	0.9935
PT10 vs. PTc10	-1.564	-2.158 to -0.9696	Yes	****	<0.0001
PT10 vs. PTc20	-0.1480	-0.7424 to 0.4464	No	ns	0.8907
PT20 vs. PTc10	-1.510	-2.104 to -0.9154	Yes	****	<0.0001
PT20 vs. PTc20	-0.09380	-0.6882 to 0.5006	No	ns	0.9684
PTc10 vs. PTc20	1.416	0.8216 to 2.010	Yes	****	<0.0001

(**c**)

Tukey's multiple comparisons test	Mean Diff.	95.00% CI of diff.	Below threshold?	Summary	Adjusted P Value
PH10 vs. PH20	1.349	0.4783 to 2.221	Yes	**	0.0021
PH10 vs. PHc10	-2.874	-3.745 to -2.003	Yes	****	<0.0001
PH10 vs. PHc20	-1.672	-2.543 to -0.8011	Yes	***	0.0003
PH20 vs. PHc10	-4.223	-5.094 to -3.352	Yes	****	<0.0001
PH20 vs. PHc20	-3.022	-3.893 to -2.150	Yes	****	<0.0001

PHc10 vs. PHc20	1.202	0.3305 to 2.073	Yes	**	0.0057
PT10 vs. PT20	-0.1148	-0.9481 to 0.7185	No	ns	0.9785
PT10 vs. PTc10	-5.991	-6.824 to -5.158	Yes	****	<0.0001
PT10 vs. PTc20	-3.929	-4.762 to -3.096	Yes	****	<0.0001
PT20 vs. PTc10	-5.876	-6.710 to -5.043	Yes	****	<0.0001
PT20 vs. PTc20	-3.814	-4.648 to -2.981	Yes	****	<0.0001
PTc10 vs. PTc20	2.062	1.229 to 2.895	Yes	****	<0.0001

(d)

Tukey's multiple comparisons test	Mean Diff.	95.00% CI of diff.	Below threshold?	Summary	Adjusted P Value
PH10 vs. PH20	1.188	0.5804 to 1.796	Yes	***	0.0002
PH10 vs. PHc10	-1.386	-1.994 to -0.7784	Yes	****	<0.0001
PH10 vs. PHc20	-1.198	-1.805 to -0.5902	Yes	***	0.0002
PH20 vs. PHc10	-2.574	-3.182 to -1.966	Yes	****	<0.0001
PH20 vs. PHc20	-2.386	-2.993 to -1.778	Yes	****	<0.0001
PHc10 vs. PHc20	0.1882	-0.4194 to 0.7958	No	ns	0.8120
PT10 vs. PT20	0.9298	0.1975 to 1.662	Yes	*	0.0108
PT10 vs. PTc10	-1.888	-2.620 to -1.155	Yes	****	<0.0001
PT10 vs. PTc20	-0.3084	-1.041 to 0.4239	No	ns	0.6326
PT20 vs. PTc10	-2.817	-3.550 to -2.085	Yes	****	<0.0001
PT20 vs. PTc20	-1.238	-1.970 to -0.5059	Yes	***	0.0009
PTc10 vs. PTc20	1.579	0.8469 to 2.311	Yes	****	<0.0001

(e)

Tukey's multiple comparisons test	Mean Diff.	95.00% CI of diff.	Below threshold?	Summary	Adjusted P Value
PH10 vs. PH20	89.52	14.03 to 165.0	Yes	*	0.0176
PH10 vs. PHc10	-335.4	-410.9 to -259.9	Yes	****	<0.0001
PH10 vs. PHc20	-196.6	-272.1 to -121.1	Yes	****	<0.0001
PH20 vs. PHc10	-424.9	-500.4 to -349.4	Yes	****	<0.0001
PH20 vs. PHc20	-286.1	-361.6 to -210.7	Yes	****	<0.0001
PHc10 vs. PHc20	138.8	63.31 to 214.3	Yes	***	0.0004
PT10 vs. PT20	36.44	-38.13 to 111.0	No	ns	0.5183
PT10 vs. PTc10	-599.5	-674.1 to -524.9	Yes	****	<0.0001
PT10 vs. PTc20	-346.3	-420.9 to -271.8	Yes	****	<0.0001
PT20 vs. PTc10	-635.9	-710.5 to -561.4	Yes	****	<0.0001
PT20 vs. PTc20	-382.8	-457.3 to -308.2	Yes	****	<0.0001
PTc10 vs. PTc20	253.2	178.6 to 327.7	Yes	****	<0.0001

Disclaimer/Publisher's Note: The statements, opinions and data contained in all publications are solely those of the individual author(s) and contributor(s) and not of MDPI and/or the editor(s). MDPI and/or the editor(s) disclaim responsibility for any injury to people or property resulting from any ideas, methods, instructions or products referred to in the content.
